# Disparities in Utilization of the World Trade Center Health Program Among World Trade Center Rescue and Recovery Workers and Volunteers

**DOI:** 10.3390/ijerph22040643

**Published:** 2025-04-19

**Authors:** Caleb D. Ayers, Rebecca D. Kehm, James E. Cone, Jiehui Li

**Affiliations:** 1 World Trade Center Health Registry, Center for Population Health Data Science, New York City Department of Health and Mental Hygiene, Long Island City, NY 11101, USA; cayers@health.nyc.gov (C.D.A.); rkehm@health.nyc.gov (R.D.K.); jcone@health.nyc.gov (J.E.C.); 2 Department of Epidemiology, Mailman School of Public Health, Columbia University, New York, NY 10032, USA

**Keywords:** disparities, disaster, World Trade Center (WTC), WTC health program (WTCHP), utilization of health program, WTC rescue and recovery workers and volunteers

## Abstract

The 11 September 2001 World Trade Center (WTC) rescue and recovery workers (RRWs) included first responders (FDNY and NYPD), volunteers, and other workers. Volunteers were often more vulnerable than first responders to adverse health outcomes resulting from the exposure. It is not yet known whether there are differences in WTC Health Program (WTCHP) utilization by worker type. This is a cross-sectional study of 20,012 WTCHP-eligible RRWs to examine whether worker type was associated with WTCHP utilization based on self-reported data from four WTC Health Registry follow-up surveys (2006–2021), using multivariable log-binomial regression adjusted for sociodemographic factors and comorbidities. We also examined factors associated with WTCHP utilization by worker type. Overall, 9584 RRWs (47.9%) reported receiving WTCHP services, but only 22.5% of volunteers reported WTCHP utilization. After adjustment, first responders and other workers were, respectively, 2.73 (95% CI = 2.56, 2.92) and 1.69 (95% CI = 1.58, 1.80) times more likely to utilize WTCHP service than volunteers. Sociodemographic factors and comorbidities were consistently associated with WTCHP utilization across worker types, except for race/ethnicity. Among those eligible, the volunteer group reported the lowest utilization of WTCHP among worker types, suggesting that WTC volunteers should be a priority group for outreach regarding access and utilization of WTCHP.

## 1. Introduction

The attacks on the World Trade Center (WTC) in New York City (NYC) on 11 September 2001 exposed an estimated 410,000 people to toxic contaminants and emotionally stressful conditions. Over 91,000 were rescue, recovery, and clean-up workers, including a wide range of individuals, from firefighters and police officers to construction and sanitation workers, from other professionals to volunteers [1,2,3].

Following the WTC attacks, efforts were made to address the health concerns of responders. The NYC Department of Health emergency operations center opened immediately after the 11 September 2001 attacks and started many activities, including establishing a surveillance system for tracking victims presenting to hospitals. The Fire Department of the City of New York (FDNY) WTC treatment program began on 11 September 2001 for FDNY personnel who responded to the 9/11 attacks [4]. The Mount Sinai School of Medicine WTC Worker Monitoring Program was established in July 2002 for affected non-FDNY responders [5]. Beginning on 1 July 2011, the CDC’s National Institute for Occupational Safety and Health (NIOSH) WTC Health Program (WTCHP) created under the James Zadroga 9/11 Health and Compensation Act of 2010, continued and expanded medical monitoring and treatment of specific 9/11-related health conditions among eligible responders and survivors [3]. More details on WTCHP eligibility, enrollment, and services are provided elsewhere [3]. Previous research has shown that there are health benefits to being enrolled in WTCHP among WTC responders. These include lower cancer-specific and all-cause mortality among WTC responders with cancer enrolled in WTCHP compared to cancer patients diagnosed in New York State who were not WTC responders [6] and significantly lower all-cause mortality risk among those WTC responders enrolled in WTCHP compared to those not enrolled [7].

The WTCHP continues to monitor and provide care for those affected by the 9/11 attack, offering services that may be especially beneficial for those most impacted by their exposure. This includes volunteers involved in the WTC rescue and recovery efforts, who have been shown to be more vulnerable to adverse health effects after exposure than first responders [8,9], although WTC responders, in general, have experienced a range of physical and mental health issues, such as respiratory problems, and post-traumatic stress disorder (PTSD) [10,11,12,13]. The WTCHP for WTC responders ensures volunteers affected by the WTC disaster have access to the healthcare support they need, similar to the first responders.

Nevertheless, little is known on whether utilization of WTCHP differed by type of WTC responders, which is important for promoting health equity among the 9/11-exposed population and may offer valuable insights for improving healthcare access in other disaster-relief settings. In the present study, we analyzed data from the WTC Health Registry to examine whether WTC worker type was associated with WTCHP utilization among eligible WTC responders identified from the WTC Health Registry cohort. We also examined whether sociodemographic factors and comorbidities were associated with WTCHP utilization within each worker type.

## 2. Materials and Methods

### 2.1. Study Population

**Figure 1:**
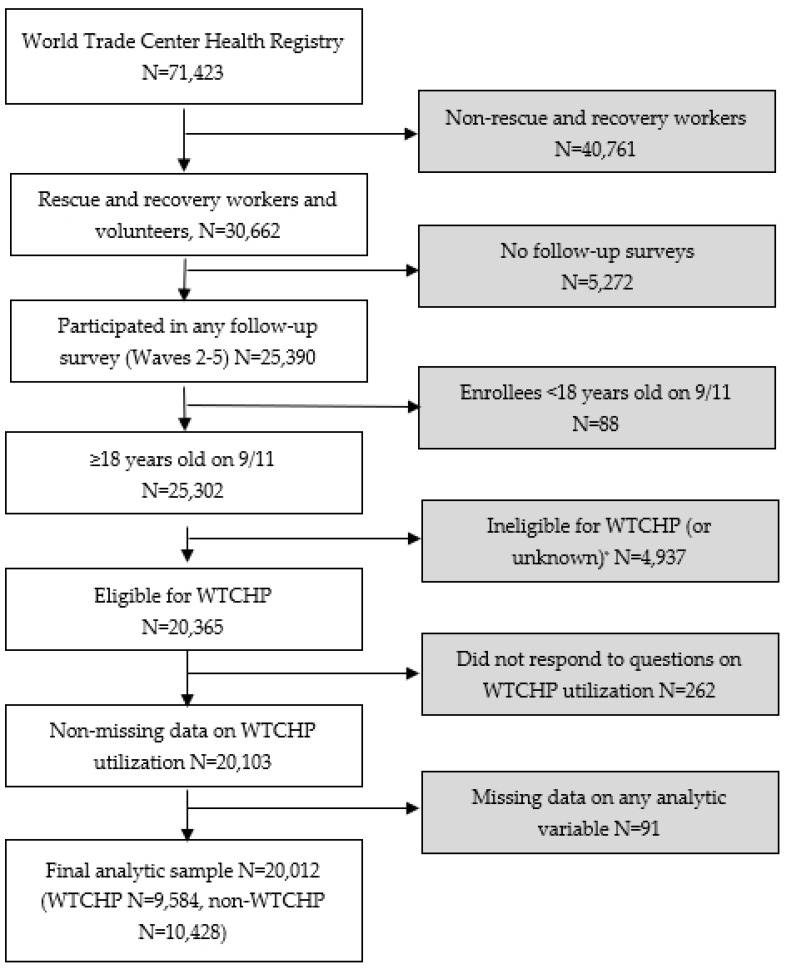
Selection of analytic sample. Abbreviations WTCHP: World Trade Center Health Program; N: number. * Included those whose only known worksite was Staten Island (N = 802) because detailed data on time period and hours were not available in WTC Health Registry surveys.

The WTC Health Registry cohort consists of 43% WTC rescue, recovery, and cleanup workers and volunteers (hereafter referred to as RRWs) and 57% survivors who were not involved in WTC rescue and recovery effort, with a total of over 71,000 enrollees responding to the Wave 1 survey in 2003–2004 (Baseline survey, Wave 1) [1]. After recruitment, four follow-up surveys have been conducted in 2006–2007 (Wave 2), 2011–2012 (Wave 3), 2015–2016 (Wave 4), and 2020–2021 (Wave 5), respectively [2,14]. For this study, we focused on RRWs in the WTC Health Registry who were eligible for enrollment into WTCHP. As shown in Figure 1, we first restricted the analytic sample of RRWs (N = 30,662) to those who participated in any follow-up survey Waves 2–5 (N = 25,390) since survey questions on WTCHP utilization were asked in each of these wave surveys. We then excluded RRWs who were less than 18 years old on 9/11 due to small numbers in this age group (N = 88), who were ineligible for WTCHP (or unknown eligibility) based on available data on date and hours of rescue and recovery work required by NIOSH for eligibility [15] (N = 4937), or who did not respond to questions on WTCHP utilization in any of the Waves 2–5 follow-up surveys (N = 262). Using Wave 1 survey data, we compared demographic characteristics between enrollees included in the study and those excluded and found no clinically meaningful differences between the two groups. Therefore, we performed a complete case analysis, which resulted in 91 more exclusions due to missing data on any analytic variable. This process resulted in a final analytic sample of 20,012 RRWs (64.9% of total number of RRWs enrolled in the WTC Health Registry).

In the present study, RRWs eligible for WTCHP included those affiliated with FDNY and the remaining RRWs who met the NIOSH eligibility criteria as determined by available data collected in Wave 1. This included non-FDNY RRWs who worked or volunteered on the WTC effort for either 4 h from 11 to 14 September 2001, 24 h in September 2001, or 80 h between 11 September 2001 to 31 July 2002; or police officers affiliated with the New York Police Department or Port Authority of New York State and New Jersey who worked at Ground Zero for 4 h between 11 September 2001 and 31 July 2002, based on NIOSH’s enrollment criteria [15].

### 2.2. Outcome: WTCHP Utilization

Starting at Wave 2, WTCHP utilization was assessed with the question, “Have you received services from any of the following 9/11-related medical monitoring or treatment programs?”; subsequent waves asked, “Have you ever received services from any World Trade Center (WTC) health program” with a list of the programs. We classified individuals as having utilized WTCHP if they responded “yes” to receiving WTCHP services on any of the Wave 2–5 surveys. We classified individuals as non-WTCHP users if they answered “no” to receiving WTCHP services on at least one of the Waves 2–5 surveys and never answered “yes” to this question.

### 2.3. Exposure Variable: Worker Type

Worker type was defined based on available data collected at Wave 1 survey and categorized as “first responder”, “volunteer,” or “other.” First responders included RRWs affiliated with FDNY and the New York Police Department. Volunteers included individuals who reported volunteering in rescue and recovery activities between 11 September 2001 and 30 June 2002. All remaining RRWs were assigned to others, which included any other worker type (e.g., sanitation, construction, and utility workers), regardless of their affiliations.

### 2.4. Covariates

Data on covariates, which were selected a priori, were all self-reported and included sex (female/male), age on 9/11, race/ethnicity (non-Hispanic White, non-Hispanic Black, Hispanic and all others), educational attainment (high school graduate or lower, some college, and college graduate or above), NYC residency (yes vs. no), WTC Health Registry recruitment source (self-identified vs. list-identified), and physician-diagnosed comorbidities (covered conditions, non-covered conditions, and none of above).

For variables that may change over time (education, NYC residency, and comorbidities), we selected data from the Wave survey in which WTCHP utilization status was determined. For WTCHP users, this was the first survey wave in which they reported using WTCHP services. For non-WTCHP users, this was the last survey wave in which they reported never using WTCHP services. If a data point was missing from the survey Wave used to determine WTCHP utilization, we used data from the most recent preceding survey with a non-missing value for the variable. NYC residency was defined as “NYC resident” versus “non-resident” at the time enrollees reported their WTCHP utilization status. Categorization of comorbidities focused on self-reported conditions that may be covered by WTCHP. This variable was defined as “covered condition(s)”, “non-covered condition(s)”, or “none of the above”. Individuals were first classified as having a covered condition(s) if they reported ever being diagnosed by a healthcare professional with at least one of the following conditions: anxiety, asbestosis, asthma, chronic bronchitis, chronic obstructive pulmonary disease, chronic sinusitis, depression, emphysema, gastroesophageal reflux disease, PTSD, pulmonary fibrosis, reactive airway disease, or substance use disorder. These conditions were included as “covered conditions” as they are medically covered by WTCHP if they meet certain NIOSH-defined criteria, such as being diagnosed within a designated time frame after 11 September 2001. Covered conditions were broadly defined in this analysis, given that we do not have related data to fully align with the NIOSH’s required maximum time interval [16]. Individuals were classified as having non-covered condition(s) if they reported a diagnosis of at least one of the following conditions: angina, coronary artery disease, diabetes, hypertension, myocardial infarction, other heart disease, or stroke. These are conditions that have not been covered by WTCHP thus far. Lastly, individuals were classified under none of the above if they did not report a diagnosis of any of the above conditions.

### 2.5. Statistical Analysis

We first calculated the response rates for each survey wave (Waves 1–5) by worker type to assess potential information bias. We then described the differences in characteristics by WTCHP utilization using a chi-squared (χ^2^) test. We examined whether worker type was associated with WTCHP utilization by calculating the crude and adjusted relative risks (aRRs) and 95% confidence intervals (CIs) estimated from log-binomial regression models. We also fitted multivariable log-binomial regression models to examine adjusted associations of the covariates with WTCHP utilization stratified by worker type. The covariates in the multivariable log-binomial models included age on 9/11, sex, race, ethnicity, education, NYC residency, comorbidities, and type of WTCHR enrollment. Statistical analyses were conducted using Statistical Analysis System (SAS) v9.4 (SAS Institute Inc., Cary, NC, USA). All statistical tests were 2-sided, and *p*-value less than 0.05 was considered statistically significant.

## 3. Results

### 3.1. Description of the Study Sample

Among a total of 20,012 RRWs included in the analysis, the proportion of RRWs who provided information on WTCHP utilization decreased with each successive survey wave (Waves 1–5), but non-response rates did not vary over time by worker type (Table 1).

A total of 47.9% (N = 9584) RRWs reported having utilized WTCHP services, of which volunteers reported the lowest use of WTCHP services (Table 2). WTCHP utilization was higher among males (51.0% versus 33.7% among females), in age 25–44 years on 9/11 (50.6% versus 28.2% and 45.2% in the 18–24 and ≥45 years age groups, respectively), non-Hispanic Whites (49.3% versus 47.9%, 40.3%, and 39.9% in Hispanics, non-Hispanic Blacks, and all other races, respectively), educational attainment below a college degree (50.5–51.6% versus 42.8% in those with a college degree or higher), NYC residents (53.0% versus 44.7% in non-residents), and those with covered conditions (52.7% versus 37.9% and 45.0% in those with non-covered conditions and neither, respectively). The prevalence of covered conditions varied by worker type, with volunteers having the highest prevalence (59.2% versus 52.7% and 54.7% in first responders and other workers, respectively). All χ^2^ tests were statistically significant (*p* < 0.001).

### 3.2. Associations of Worker Type and Covariates with WTCHP Utilization 

**Figure 2:**
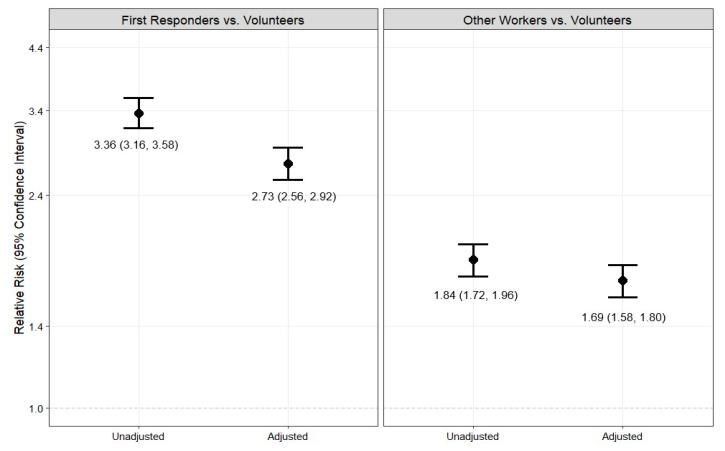
Unadjusted and adjusted relative risks and 95% confidence intervals for the association of worker type with WTCHP utilization in the World Trade Center Health Registry, N = 20,012. Legend: Adjusted relative risks and 95% confidence intervals are adjusted for age on 9/11, sex, race, ethnicity, education, NYC residency, comorbidities, and type of WTC Health Registry enrollment. First responders include rescue/recovery workers who were affiliated with Fire Department of New York City or New York City Police Department. Abbreviations: WTCHP: World Trade Center Health Program.

**Figure 3:**
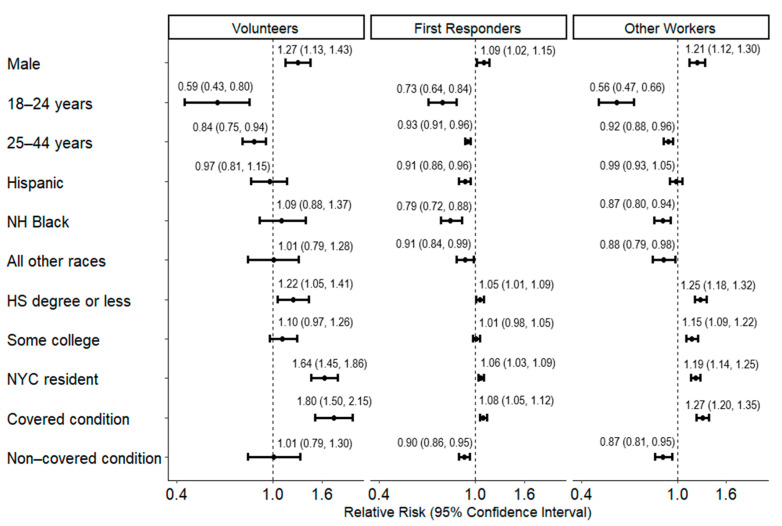
Adjusted relative risks and 95% confidence intervals for the associations of sociodemographic characteristics and medical conditions with World Trade Center Health Program utilization by worker type, World Trade Center Health Registry, N = 20,012. Legend: Relative risks and 95% confidence intervals are adjusted for the other variables in the figure and type of WTC Health Registry enrollment. Refer to Table 1 to identify the reference category for each variable (e.g., ≥45 years is the reference category for age on 9/11). First responders include rescue/recovery workers who were affiliated with Fire Department of New York City or New York City Police Department. Covered condition includes at least one reported diagnosis of anxiety, asbestosis, asthma, chronic bronchitis, chronic obstructive pulmonary disease, chronic sinusitis, depression, emphysema, gastroesophageal reflux disease, post-traumatic stress disorder, pulmonary fibrosis, reactive airway disease, or substance use disorder, which are all covered by World Trade Center Health Program. Uncovered conditions include at least one reported diagnosis of angina, coronary artery disease, diabetes, hypertension, other heart conditions, myocardial infarction, and stroke, which are all conditions not covered by WTCHP after classifying those with a covered condition. Abbreviations: HS = high school; WTCHP = World Trade Center Health Program; NH = non-Hispanic.

After adjusting for covariates, first responders and other workers were 2.73 (95% CI = 2.56–2.92) and 1.69 (95% CI = 1.58–1.80) times more likely to utilize WTCHP services, respectively, compared to volunteers (Figure 2). In the multivariable analysis stratified by the three worker types, being male, being older in age, residing in NYC, having lower educational attainment, and having covered conditions were significantly associated with WTCHP utilization across worker types (Figure 3).

Notably, among volunteers, individuals with a covered condition were 80% more likely to utilize WTCHP compared to those without any condition (RR = 1.80, 95% CI = 1.50–2.15). In contrast, among first responders and other workers, individuals with a covered condition were 8% (RR = 1.08, 95% CI = 1.05–1.12) and 27% (RR = 1.27, 95% CI = 1.20–1.35) more likely, respectively, to utilize WTCHP than those without any condition (Figure 3). Among first responders, Hispanic (RR = 0.91, 95% CI = 0.86–0.96) and non-Hispanic Black responders (RR = 0.79, 95% CI = 0.72–0.88) were less likely to report WTCHP utilization compared to non-Hispanic White responders. Among other workers, non-Hispanic Black (RR = 0.87, 95% CI = 0.80–0.94) and other race/ethnicity (RR = 0.88, 95% CI = 0.79–0.98) workers were less likely to report WTCHP utilization compared to being non-Hispanic White workers. Race/ethnicity was not associated with WTCHP utilization among volunteers.

## 4. Discussion

This is the first study to investigate whether worker type is associated with self-reported WTCHP utilization among WTCHP-eligible RRWs. We found that the volunteer group has the lowest proportion (22.5%) of individuals utilizing WTCHP services among the three worker types examined. Multivariable analyses supported that lower WTCHP utilization in volunteers compared to first responders and other workers was not accounted for by differences in sociodemographic characteristics or comorbidities. Additionally, when we examined whether sociodemographic characteristics and comorbidities were associated with WTCHP utilization by worker type in multivariable analysis, we found consistent patterns of association across groups for most factors. Specifically, male sex, older age on 9/11 (≥45 years old), NYC residency, lower educational attainment, and having covered conditions were significantly associated with WTCHP utilization across the three worker types. However, race/ethnicity was not associated with WTCHP utilization in the volunteer group, whereas non-Hispanic white race/ethnicity in the other two worker groups was associated with increased WTCHP utilization. Taken together, these findings suggest that volunteers are a target group for WTCHP outreach, and targeted outreach based on certain factors like race/ethnicity may need to be specific to each worker type.

The present study found that volunteers, in addition to having the lowest rate of WTCHP utilization, also had the highest prevalence of health conditions that are covered by WTCHP compared to other worker groups. Previous studies have also found that volunteers are more likely to have adverse health outcomes compared to other worker types [8,9]. A previous study of WTC Health Registry RRWs reported that the overall prevalence of PTSD among RRWs at the time of enrollment in the WTC Health Registry was 12.4%, with rates varying by worker type, from 6.2% among police officers to 21.2% among unaffiliated volunteers [8]. A subsequent report from the same study cohort, focusing on those who responded to the Wave 2 survey, found that unaffiliated volunteers at the WTC site experienced a higher burden of 9/11-related exposure and adverse health outcomes compared to affiliated volunteers [9]. This included more intense dust cloud exposure, a higher number of witnessed horrific events, more injuries sustained on 9/11, higher unmet healthcare needs, early post-9/11 mental health diagnoses, asthma, PTSD, and lower respiratory symptoms [12]. Due to a lack of or inadequate training and preparation for the physical and mental demands of disaster response, as well as limited post-disaster social support and healthcare resources, volunteers may be more vulnerable to adverse health outcomes compared to professional responders [9,17]. Therefore, given the findings of this study, efforts to improve volunteers’ enrollment and utilization of WTCHP should be highlighted and prioritized.

Our evaluation of other characteristics related to WTCHP utilization within each worker type provides additional direction for future studies of the WTCHP program. For example, our findings related to comorbidities deserve special attention due to the potential for surveillance bias. Specifically, we found that those with covered conditions were more likely to utilize WTCHP across all worker types. This could be due to those with healthcare needs potentially covered by the program appropriately accessing care, or it could be due to increased diagnostic identification of covered conditions after WTCHP enrollment. Unfortunately, we are not able to test these hypotheses because we lack data on the timing of the diagnosis of covered conditions in relation to the utilization of WTCHP. Nevertheless, our finding that almost half (47.3%) of RRWs eligible for WTCHP (i.e., meeting NIOSH enrollment criteria based on WTC exposure) who reported having a likely covered condition also reported never utilizing WTCHP services highlights the urgent need for targeted outreach efforts to connect RRWs with covered conditions to the care and benefits available through the WTCHP. Additionally, although it is not yet sensible to target those with non-covered conditions (treatment for their conditions is not covered by the program), there could be benefits to research and initiatives focused on better understanding these conditions in the WTC-exposed population. For example, there are studies linking 9/11 exposure to adverse cardiovascular outcomes, which are not currently covered by WTCHP [18,19,20,21].

The main strength of this study was the large sample of 9/11 RRWs and multiple surveys that enabled us to capture and assess WTCHP utilization over an extended period across different worker types. The main study limitation was the reliance on self-report to identify WTCHP utilization. This may have led to potential response and information bias, especially in individuals who did not complete each survey wave. However, participation in each Wave by worker type remained stable over time (Table 1), suggesting nondifferential misclassification of exposure (i.e., WTCHP utilization) by worker type. We also note that the wording of the WTCHP utilization question slightly changed between Waves 2 and 3, which may have introduced minor differences in how participants interpreted or responded to the question. There was also limited information on the frequency of WTCHP utilization, the type of services used, and the timing of WTCHP utilization in relation to the diagnosis of the covered condition. Therefore, we were not able to establish a temporal or causal relationship between the diagnosis of covered conditions and WTCHP utilization. Lastly, we used a broader definition of “covered conditions” than NIOSH [16], given that we were not able to establish the time interval between 9/11 and diagnosis because we lacked clinical data (e.g., the initial onset of symptoms for aerodigestive disorders). As a result, some conditions may have been misclassified as covered, even though their diagnosis occurred outside the defined time frame relative to 9/11. Therefore, studies with clinical data are needed to explore this relationship further. Despite these limitations, this study provides important insights and future directions for increasing WTCHP utilization among RRWs.

## 5. Conclusions

This study found that volunteers are less likely to utilize WTCHP services than the other types of RRWs. This is concerning, especially given that volunteers have already been identified as a vulnerable population at increased risk for adverse health outcomes associated with 9/11-related exposures. Further research is needed to understand barriers to accessing WTCHP services among volunteers, and future outreach efforts should prioritize this particular group of RRWs to promote health equity within the WTC-exposed population. More broadly, these findings highlight the need for ongoing monitoring and support for volunteers involved in other disaster-relief efforts, ensuring they receive the same level of medical care and resources as other responders.

## Figures and Tables

**Table 1:** World Trade Center Health Registry survey participation in each Wave during 2003–2020 by worker type.

Worker Type	Wave 1		Wave 2		Wave 3		Wave 4		Wave 5	
	N	Col %	N	Col %	N	Col %	N	Col %	N	Col %
Total	20,012	100	17,165	100	16,019	100	13,636	100	10,675	100
Volunteer	3611	18.0	3089	18.0	2842	17.7	2378	17.4	1724	16.2
First responder ^a^	7075	35.4	6207	36.2	5755	35.9	5096	37.4	4148	38.9
Other	9326	46.6	7869	45.8	7422	46.3	6162	45.2	4803	45.0

Abbreviations: Col = column; N = number. ^a^ Included WTC rescue/recovery workers who were affiliated with Fire Department of New York City or New York City Police Department.

**Table 2:** Characteristics of rescue and recovery workers by WTCHP utilization among eligible rescue/recovery workers in the World Trade Center Health Registry (N = 20,012).

Characteristic	WTCHP		Non-WTCHP	
	N	Row %	N	Row %
Total	9584	47.9	10,428	52.1
Gender				
Male	8382	51.0	8058	49.0
Female	1202	33.7	2370	66.3
Age on 9/11, years				
18–24	222	28.2	565	71.8
25–44	6239	50.6	6079	49.4
≥45	3123	45.2	3784	54.8
Race/ethnicity				
Non-Hispanic White	7224	49.3	7427	50.7
Non-Hispanic Black	646	40.3	956	59.7
Hispanic	1285	47.9	1400	52.1
All other	429	39.9	645	60.1
Education Attainment				
High school graduate/GED or less	2664	50.5	2611	49.5
Some college	3590	51.6	3364	48.4
College graduate or above	3330	42.8	4453	57.2
Type of WTC Health Registry enrollment				
Self-identified	7800	54.9	6395	45.1
List-identified	1784	30.7	4033	69.3
New York City residence ^a^				
NYC	4082	53.0	3624	47.0
All other	5502	44.7	6804	55.3
Comorbidities				
Covered condition(s) ^b^	5793	52.7	5206	47.3
Non-covered condition(s) ^c^	1404	37.9	2303	62.1
None of the above	2387	45.0	2919	55.0
Worker type				
Volunteer	812	22.5	2804	77.5
First responder ^d^	4425	75.4	1441	24.6
All Others	4347	41.3	6183	58.7

Abbreviations: GED = General Education Diploma; N = number; WTCHP = World Trade Center Health Program. ^a^ NYC residence refers to the residence at the time enrollees reported their WTCHP utilization status. ^b^ At least one reported diagnosis of anxiety, asbestosis, asthma, chronic bronchitis, chronic obstructive pulmonary disease, chronic sinusitis, depression, emphysema, gastroesophageal reflux disease, post-traumatic stress disorder, pulmonary fibrosis, reactive airway disease, or substance use disorder (all covered by WTCHP). ^c^ At least one reported diagnosis of angina, coronary artery disease, diabetes, hypertension, other heart conditions, myocardial infarction, and stroke (not covered by WTCHP) after classifying those with a covered condition. ^d^ Included rescue/recovery workers who were affiliated with Fire Department of New York City or New York City Police Department.

## Data Availability

World Trade Center Health Registry data may be made available following review of applications to the Registry from external researchers. The data are not publicly available due to privacy or ethical restrictions.

## Abbreviations

The following abbreviations are used in this manuscript:

aRRs	Adjusted relative risks
CI	Confidence interval
FDNY	Fire Department of New York
RRWs	Rescue and recovery workers and volunteers
WTCHP	World Trade Center Health Program
NIOSH	National Institute for Occupational Safety and Health
NYC	New York City
NYPD	New York City Police Department
PTSD	Post-traumatic stress disorder
